# Treatment of lumbar brucella spondylitis with negative pressure wound therapy *via* extreme lateral approach: A case report

**DOI:** 10.3389/fsurg.2022.974931

**Published:** 2022-10-26

**Authors:** Haocheng Cui, Zhengqi Chang, Xiuchun Yu

**Affiliations:** Department of Orthopedics, 960th Hospital of PLA, Jinan, China

**Keywords:** brucella spondylitis, vacuum sealing drainage (VSD), negative pressure wound therapy (NPWT), extreme lateral interbody fusion (XLIF), spinal infection

## Abstract

Brucella spondylitis (BS) is a specific spinal infection. Surgical treatment is required for Brucella spondylitis that has caused neurological symptoms in the lower extremities and developed an intraspinal abscess. The main purpose of surgery is to remove the lesion and restore the stability of the spine. However, both the anterior approach and the posterior approach cannot completely remove the lesions, resulting in a low cure rate and a certain recurrence rate. Although anterior or posterior debridement is more thorough, it is unbearable for some patients with poor general condition. In this study, for the first time, a negative pressure wound therapy (NPWT) device was introduced into the intervertebral space through the extreme lateral approach to treat a patient with Brucella spondylitis. We summarize the treatment process, and discuss the feasibility and effectiveness of this surgical approach through 1-year follow-up.

## Introduction

Brucella spondylitis (BS) is a spinal infection caused by Brucella infection of the vertebral body or intervertebral disc, among which the lumbar spine is the most susceptible (1). The treatment of lumbar Brucella spondylitis is exceedingly complex due to the anatomical characteristics of the disc tissue. Although antibiotic treatment is the most critical and vital aspect, insufficient blood flow and the ease of infection spread render it ineffective (2). The purpose of surgical treatment is to remove the lesion, alleviate the patient's symptoms, preserve and reconstruct the stability, and relieve the compression. However, due to the severe infection of the lumbar spine, the lesions are surrounded by vital and complicated structures, making surgical therapy extremely challenging. Whether anterior, posterior, or mixed approach, debridement is difficult to be as straightforward and comprehensive as with limb bone infection, and the therapy impact is greatly diminished.

Extreme lateral interbody fusion (XLIF), as a mature spinal surgery approach technique in recent years, has been widely used in lumbar spinal stenosis, lumbar instability, degenerative scoliosis and lumbar spine infections (3–7). XLIF has several advantages in the treatment of spinal infections. Li et al. (8) applied XLIF technology to treat 13 patients with lumbar spine infection in phase 1, including 8 cases of non-specific infection, 4 cases of tuberculosis, and 1 case of brucellosis. All 13 cases had single-level lumbar intervertebral space involvement, the average operation time was 90 min, and the average intraoperative blood loss was 70 ml. Nerve electrophysiological monitoring was used during the operation, and there was no nerve, blood vessel, or organ injury after operation. The VAS and ODI scores of the patients after operation were significantly lower than those before operation. All patients were followed up for more than 12 months, and there was no recurrence of infection. This result gives a very positive assessment of the efficacy of XLIF in the treatment of lumbar spine infections. Wang et al. (9) treated 22 patients with lumbar spine tuberculosis through an extremely lateral approach combined with lateral or posterior percutaneous screw fixation. It is concluded that the extreme lateral approach is a minimally invasive and effective method for the treatment of spinal infection.

In this study, for the first time, an NPWT sponge was introduced into the intervertebral space of a patient with a lumbar brucellosis infection using an extreme lateral surgical approach, with positive therapeutic results.

## Case presentation

**Figure 1:**
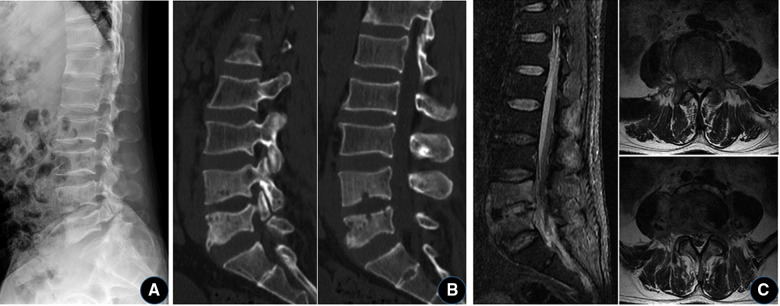
A 40-year-old man with Brucella lumbar infection and bilateral isthmus of the L5 vertebral body. (**A**) Plain x-ray shows degeneration of the lumbar spine, with a slight narrowing of the L4/5 intervertebral space and bilateral isthmus rupture at L5. (**B**) CT showed “worm-eaten-like” bone destruction in the L4 and L5 vertebral bodies, and L5 lumbar spondylolysis. (**C**) MRI showed abnormal signal in the L4/5 vertebral body and space, and the formation of abscess in the spinal canal.

A 40-year-old male patient presented to our department because of “recurrent low back pain for half a year”. The patient had a history of contact with livestock such as sheep and cattle. The physical examination revealed a limp and lower back pain with percussion pain in the lower back, but no evidence of radiculopathy. The hypoesthesia on the front of the left knee and inner calf. The left knee tendon reflex was not elicited, and the right knee tendon reflex was normal. Achilles tendon reflexes were present bilaterally. Plain radiographs of the lumbar spine showed degeneration of the lumbar spine, slight narrowing of the L4/5 intervertebral space, and L5 lumbar spondylolysis. CT showed “worm-eaten” bone destruction in both L4 and L5 vertebrae bodies, and L5 bilateral lumbar spondylolysis. MRI showed low T1, high and low T2 mixed signals in the L4, L5 vertebral bodies and L4/5 intervertebral space, and patchy high and low mixed signals in the spinal canal behind the L5 vertebral body. This result indicated spinal abscess formation (Figure 1). The results of laboratory tests showed elevated erythrocyte sedimentation rate (ESR) and C-reactive protein (CRP), and decreased hemoglobin and albumin. Blood microbial culture found positive for Brucella. Brucella agglutination test: positive. The initial diagnosis was: (1) Brucella lumbar infection, (2) L5 vertebral bilateral isthmus. After admission, the patient received full course, combined antibiotic therapy: doxycycline (100 mg, oral, bid, 3 months) + gentamicin (5 mg/kg, intramuscular injection, Qd, 1 week) + rifampicin (10 mg/kg, up to 900 mg orally, 3 months). Then, under general anesthesia, the lumbar vertebral lesion debridement + NPWT was performed through the extreme lateral approach.

**Figure 2:**
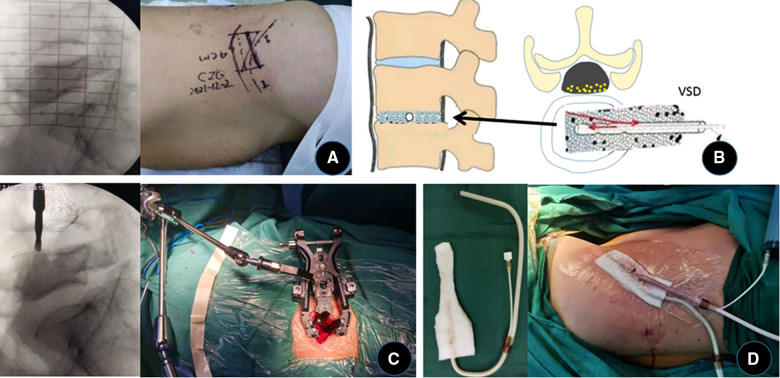
Lumbar lesion debridement + VSD drainage through the extreme lateral approach. (**A**) Locate and mark the body surface (there is a problem with the marking time, and the re-examination is less than 1 year). (**B**) Schematic of VSD placement. (**C**) After separation of the psoas major muscle, the C-arm fluoroscopy was performed again to determine the gap, and the channel and distraction device were installed. (**D**) The VSD sponge was trimmed and placed in the intervertebral space, and another VSD sponge was covered on the body surface.

During the operation, the patient was placed in the right lateral decubitus position with the waist elevated. C-arm fluoroscopically locates the L4/5 level and marks it on the body surface. The surgical area was sterilized and draped, and a 5cm-long surgical incision was made at the projection of the left waist L4/5 space. Perform blunt dissection of the external oblique muscle, internal oblique muscle, and transverse abdominis muscle fiber direction in turn to expose the peritoneum. First, blunt dissection of the retroperitoneum to expose the psoas major muscle, and then blunt dissection along the direction of the anterior 1/3 of the psoas major muscle to reveal the vertebral body and L4/5 intervertebral space. C-arm fluoroscopy was performed again to confirm the level, the expansion channels were placed in sequence, and the automatic spreader was installed and fixed. The annulus fibrosus was incised, the inflammatory and necrotic tissue in it was thoroughly scraped off, and the intervertebral space was repeatedly washed with chlorhexidine solution (1:2,000) and normal saline. A large VSD sponge was trimmed appropriately and placed in the L4/5 intervertebral space. The body surface is covered with another piece of VSD sponge, and the negative pressure is connected after the film is sealed. The operation was ended after the drainage was smooth and the VSD device was well sealed. The intraoperative blood loss was about 80 ml without blood transfusion (Figure 2). The negative pressure of NPWT was set at 16.6–60 kpa after the operation, and the external sponge was kept contracted and sealed. The total drainage volume recorded 11 days after surgery was 630 ml.

**Figure 3:**
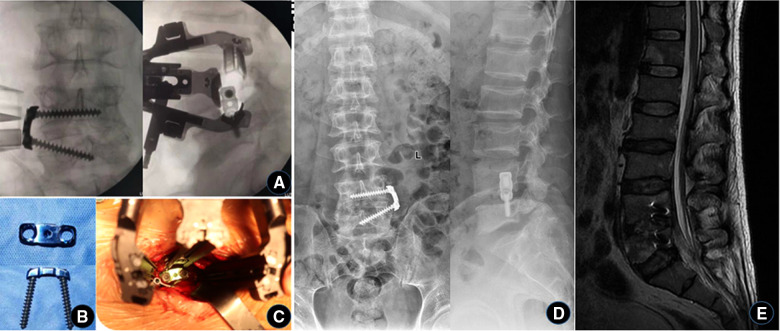
The third stage of iliac bone grafting and internal fixation. (**A–C**) During the operation, the iliac bone block was taken from the original incision, placed in the intervertebral space, and then fixed with a self-designed lateral lumbar spine plate. (**D**) X-ray and (**E**) MRI were reviewed 1 week after operation, showing that the internal fixation was well in place and the abscess in the spinal canal disappeared.

Twenty-one days after first operation, under general anesthesia, the iliac bone was taken out of the original incision and the lateral plate internal fixation was implanted, and the position was the same as the previous operation. After the VSD device was removed, the wound surface of the intervertebral space was fresh, the surrounding granulation tissue grew extensively, and there was no inflammatory exudation. The iliac was exposed free from the original incision, and an appropriate size of autologous iliac bone block and enough cancellous bone were chiseled with an osteotome. Then the L4/5 gap was exposed, the autologous iliac bone block and cancellous bone were implanted into the gap, a lateral lumbar spine fixation plate was placed, and two locking screws were screwed in for fixation. After the C-arm fluoroscopy shows that the position is good, the operation is finished by flushing. One week after the operation, MRI of the lumbar spine showed that the abscess in the spinal canal had disappeared and the implants were in proper position (Figure 3).

## Results

**Figure 4:**
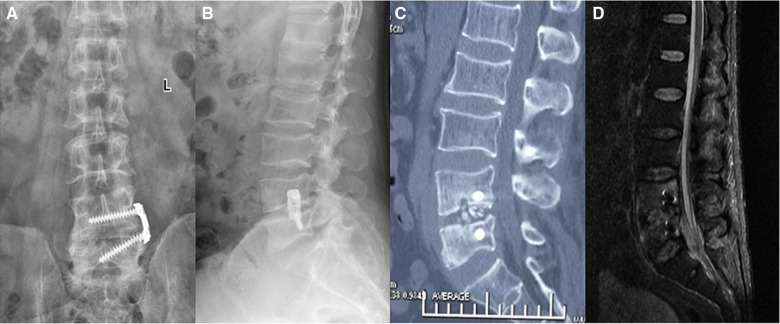
12 months postoperatively. X-rays (**A,B**), CT (**C**), and MRI (**D**) showed that the internal fixation position was good, the bone graft was fused, and the abnormal signals in the lumbar vertebral body, spinal canal, and psoas major muscle had disappeared.

After the patient was diagnosed with lumbar brucellosis infection, he was given a combination of antibiotics and a full course of treatment, including doxycycline (100 mg, oral, bid, 3 months), gentamicin (5 mg/kg, intramuscular injection, Qd, 1 week), rifampicin (10 mg/kg, up to 900 mg, orally, 3 months). ESR and CRP returned normal 3 days after the third operation. The patient was discharged after 31 days of admission. The symptoms of low back pain have completely disappeared, and he can get out of bed and walk independently. x-ray, CT, and MRI 6 months after the operation showed that the internal fixation position was good, the bone graft was fused, and the abnormal signals in the lumbar vertebral body, spinal canal, and psoas major muscle had disappeared (Figure 4). ESR and CRP were normal. The VAS score decreased from 8 points before operation to 0 points at discharge, and the ODI functional scores at 10 days, 6 months, and 1 year after operation were 42, 23, and 6 points, respectively.

## Discussion

The incidence of spondylitis in brucellosis is about 2%–53%. The lumbar vertebrae are most frequently affected, especially at L4/L5 level (1). Most patients had epidemiological contact history. Shen et al. (2) reported that about 96% (64/67) of the patients with lumbar brucella spondylitis had a clear history of livestock contact. The clinical manifestations were nonspecific, mainly including low back pain and afternoon fever, with or without lower limb neurological symptoms. The imaging manifestations are different according to the different course of the disease. During the early and middle changes, there is no obvious abnormality in x-ray film and CT, while MRI shows inflammatory infiltration of vertebral or intervertebral space, showing T1 low signal, T2 high signal or high and low mixed signal. Following the onset of the disease, x-ray, CT, and MRI scans reveal obvious positive findings. The gold standard for identifying bacterial infections, including brucellosis, is blood culture, however its sensitivity ranges from 17% to 85%, depending on the strain involved, disease stage, and antibiotic treatment history of the patient (3). Brucella agglutination test has a high positive detection rate, simple operation and low cost, but false negatives may occur. It is mainly used in the primary screening test (4). In this study, the patient has a clear history of contact with animals, with low back pain more than half a year, accompanied by neurological symptoms of the lower extremities. The imaging manifestations are bone destruction, narrowing of the intervertebral space, formation of abscess in the spinal canal, and positive Brucella agglutination test. Brucella was found in bacterial culture after first operation. Pathological biopsy revealed inflammatory necrotic tissue and proliferative granulation tissue. Combined with this clinical symptoms, signs, epidemiological contact history, serological test, etiological test and radiology results, the patient was definitely diagnosed as Brucella spondylitis.

NPWT is frequently utilized in numerous surgical disciplines. Currently, it is frequently used to treat a variety of acute, chronic, and infected wounds. Surgeons have, in fact, recognized the clinical effect. Inspired by the reliable efficacy of NPWT in the treatment of the above bone and soft tissue infections, we innovatively placed NWPT into the intervertebral space of primary lumbar infection. We believe that using NWPT to treat lumbar spondylitis can stabilize the intervertebral space lesion's environment. Due to the unique anatomical position of intervertebral lesions, thorough debridement cannot be performed as with extremity bone infection lesions. On the basis of restricted debridement, NPWT can continually suction out pus and remaining tissue exudate from the lesion using negative pressure. Simultaneously, the huge, not-too-tough residues and secretions are split and molded into granules to be suctioned away, which eliminates the bacterial adhesion focal point. NPWT simultaneously lowered bacterial burden. The number and kind of bacteria are one of the elements that determine the therapeutic efficiency of wound treatments for bone infections. Neither surgical debridement nor medical antibiotics are effective in reducing bacterial load in bone infection wounds due to the presence of bacterial biofilm (BBF). The formation of bacterial biofilms is an important reason for the failure of current antibiotic therapy (10). BBF is composed of three basic components, the bacteria itself, the extracellular matrix produced by it, and the surface liquid or liquid-air interface, and its water content can be as high as 97% (11). The NPWT pressure evenly distributes the surface of the polyvinyl alcohol (PVA) material to form an all-round drainage. PVA has good water permeability and does not hinder the passage of liquids and small particles (12). Therefore, NPWT can drain the water in the BBF through negative pressure, destroy the bacterial growth environment, lead to the necrosis of bacteria in the biofilm, and eliminate the dead space at the same time (13). The NPWT device has both space occupation and protection functions. The PVA material of NPWT is a solid polyvinyl alcohol material, which also has a certain space-occupying effect on the basis of being compressible. The PVA material is placed in the intervertebral space, which is convenient to accurately find the position of the space in the later stage and ensure sufficient space for bone grafting. In addition, the PVA foam material wraps the drainage tube, and the surrounding tissues and organs cannot touch the drainage tube, avoiding the surrounding tissue of the wound. In particular, the nerve fibers within the spinal canal, which are aspirated, lead to neurological symptoms of the lower extremities (14).

The dissection and exposure of the extreme lateral approach through the retroperitoneum can reduce traction on the peritoneum, major arteries, and nerves compared to conventional anterior and posterior approaches. Additionally, the anterior annulus of the disc and anterior longitudinal ligament are difficult to damage, which can significantly reduce the difficulties associated with conventional anterior and posterior surgery (3). Particularly for the surgical treatment of lumbar vertebra infection, the extreme lateral approach can directly reach and thoroughly expose the lesion space and vertebral body. Simultaneously, the spinal canal, spinal cord, and dura can be avoided, making the surgery safer (15). During this surgery, we directly exposed the intervertebral space of the lesion with a highly lateral approach, without harming the facet joints and compromising the spine's stability. Also because of this, two-stage lumbar fusion can be performed. After removing the lesion tissue *via* an extreme lateral approach, as opposed to a transforaminal technique, we can put a larger and more suited NPWT sponge for a more comprehensive and secure drainage effect. In addition, the L4/5 lesion space was present in this patient, and the surgical incision was made above the iliac spine. During the third phase of bone grafting and internal fixation, no additional incision was made to remove the iliac bone. Through the surgical incision of the extreme lateral approach, the iliac spine can be revealed, and it can be exposed slightly downward. It is quite convenient to use the patient's own iliac bone.

To our knowledge, there are no reports of NPWT devices inserted into the intervertebral space for the treatment of spinal infections. Based on the analysis of this patient, we believe that it is feasible to implant the NPWT device through the extreme lateral approach and two-stage lumbar fusion in the treatment of lumbar-specific infection, and the short-term follow-up effect is definite. However, further observations are needed for mid- and long-term follow-up, complications, and recurrence.

## Data Availability

The original contributions presented in the study are included in the article/Supplementary Material, further inquiries can be directed to the corresponding author/s.
